# Physiochemical Quality and Sensory Characteristics of *koji* Made with Soybean, Rice, and Wheat for Commercial *doenjang* Production

**DOI:** 10.3390/foods9080975

**Published:** 2020-07-23

**Authors:** Hyun Hee Hong, Mina K. Kim

**Affiliations:** Department of Food Science and Human Nutrition, and Fermented Food Research Center, Jeonbuk National University, 567 Baekjedaero, Deokjin-gu, Jeonju-si, Jeonbuk 54896, Korea; hhh2439876@naver.com

**Keywords:** *koji*, physiochemical quality characteristics, descriptive analysis, flavor

## Abstract

The purpose of this study was to compare the physiochemical quality characteristics and sensory profiles of three types of *koji*: soybean, rice, and wheat *koji*. *Koji* is made by inoculating *Aspergillus oryzae* following the standard method of manufacturing. The physiochemical characteristic and sensory profiles were performed after fermenting samples of *koji* for a 72 h period. The physiochemical quality characteristics that were tested include pH, moisture content, color, acidity, TA, amino-type nitrogen content, reducing- and total-sugar content, and alcohol content; the enzymatic activities that were tested include amylase (α- and β-) and protease (neutral and acidic) activities. A descriptive sensory analysis was conducted on three types of *koji* with a highly trained sensory panel (*n* = 7) using the Spectrum^TM^ Method. Differences in physiochemical and sensory profiles were observed on three *koji* samples (*p* < 0.05). Soybean *koji* had higher values in acid and TA, while rice *koji* had the highest values in reducing and total sugar, at 90.3 mg/g and 107.5 mg/g respectively. Wheat *koji* had the lowest values in protease activities. The flavor profile of soybean *koji* was characterized by bean sprout, cracker, and *cheonggukjang* aromatics; that of rice *koji* was characterized by overripe banana, solvent, syrup, and parboiled rice aromatics; and that of wheat *koji* was characterized by woody and roasted aromatics.

## 1. Introduction

*Doenjang*, fermented soybean paste, is a traditional Korean food ingredient. While the traditional *doenjang*-making process depends heavily on fermentation, companies have developed rapid fermentation methods for commercially available, mass-produced *doenjang* [1,2]. One method of manufacturing commercial *doenjang* is by using *koji*, which is made by inoculating *Aspergillus oryzae* in grainy materials such as soybean, rice, and wheat, and fermenting it for 48 to 72 h. Once the *koji* is well fermented, it is mixed with boiled soybean and fermented another month [3]. Using *koji* in *doenjang*-making can speed up the fermentation process. While traditional method takes about six months to several years to make *doenjang*, the commercial method using *koji* only takes one month to complete [1]; thus, food companies preferred to use *koji* for their *doenjang* manufacturing process.

*Koji* primarily serves as a starter for fermented foods and is widely used in the manufacturing of various Asian fermented foods in addition to *doenjang*, such as soy sauce, miso, sake, *mirin*, and *gochujang* (red pepper paste) [4,5]. *Koji* naturally contains large quantities of enzymes such as amylase and protease produced by molds, which convert starch and protein into fermented sugar and amino acids [6,7]. During protein-degradation process and the conversion of starch into sugar, the versatile flavors of *doenjang* are also developed. Therefore, the final food products using *koji* are greatly influenced by the qualities of *koji* [8]. While *nuruk*, another category of starter culture in fermented foods in Korea, is composed of various microorganisms naturally present in the environment, *koji* is made by a single microorganism under controlled fermentation conditions [9].

In order to develop the commercialized *doenjang* using *koji*, various studies were previously conducted in order to optimize the fermentation condition of *koji* and *doenjang* made with *koji*. For example, the optimum conditions of enzymatic activities including α-amylase, in *koji* made with rice, barley was reported [7]. Their study reported that increases of α-amylase activity was observed during exponential growth stage of *A. oryzae*, and decrease of enzymatic activities when *A. oryzae* reaches the stationary growth phase. Their work utilized *A. oryzae* in steamed rice and barley and fermented for 118 hrs at 28–32 °C with controlled relative humidities. Similarly, changes of enzymatic activities bythe growth of *Aspergillus oryzae S.* during soybean-based *koji* fermentation was investigated previously, and their study reported that increase of pH caused by enzymatic activities such as protease and amylase activities of 84.4 and 200 U/g, during fermentation [5]. The changes in quality characteristics of *doenjang* prepared with soybean *koji* during fermentation were investigated and reported that *Doenjang* made with soybean *koji* in which prepared with *Bacillus subtillus* 3-B-1 and *Aspergillus oryzae* 6-M-1 can provide sensory characteristics similar to that of traditionally prepared *doenjang* [10]. Earlier than their study, changes of biochemical properties and enzymatic activities of soy sauce prepared with mixture of soybean and wheat flour *koji* were reported [11]. Besides enzymatic activities and physiochemical quality activities of *koji* and *doenjang* with *koji*, analysis of functionality of *koji* was also reported such as isoflavone transformation during soybean *koji* preparation process and subsequent *doenjang* fermentation process [12].

The above-mentioned studies focused on the final products that contain *koji*, instead of investigating the quality of *koji* itself. Considering the influence of *koji* on the flavor of the final products in which it is used, it is necessary to comprehensively determine the characteristics of *koji*. To date, studies focusing on *koji* made with different sources of starchy materials have not been well documented. Therefore, the purpose of this study was to determine the physiochemical qualities and sensory characteristics of *koji* prepared with three different ingredients: soybean, rice, and wheat.

## 2. Materials and Methods

### 2.1. Koji Sample Preparation

*Koji* samples were prepared according to the standard manufacturing method used by previous studies [13,14]. Three different ingredients for *koji* preparation were selected: soybean, rice, and wheat flour (Beksul^®^ soft flour, CJ CheilJedang, Yangsan, Korea). These ingredients were purchased from a local grocery store in Jeonju, Jeollabuk-do, Korea. *Aspergillus oryzae* in powder form (Suwon Fermentation Food Research Institute, Seongnam, Korea) was purchased at a food market in Yeswine, Boen, Korea. For the preparation of soybean and rice *koji*, soybean and rice were rehydrated at room temperature for 12 h while wheat flour was thoroughly blended with 20% (w/v) distilled water. Once fully hydrated, all were autoclaved (LAC-5060S, Lab Tech, Namyangju, Korea) for 40 min at 121 °C at 0.1 mpa. Then, the soybean and rice were finely blended using a food processor (NINJA BL682KR, Hai Xin Technology Company, Shenzhen, China) at 19,354× *g* for one minute, and all equipment used for blending was prepared aseptically in order to minimize the introduction of undesirable microorganisms. Then, 0.2% (*w*/*w*) of *Aspergillus oryzae* powder was inoculated to the soybean, rice, and wheat powder and incubated at 35 °C for 72 h. All the *koji* samples were mixed using a sterilized spoon every 24 h to aerate the *koji* mixture. The *koji* was stored at a temperature of −80 °C for further analysis. Koji samples were produced three times independently.

### 2.2. Physiochemical Quality Characteristic Analyses

Physicochemical quality characteristics were analyzed following the standard methods of analysis in the Korean Food Standard Codex and a previous study [10,14], with minor modifications. All chemical compounds were purchased from the chemicals company Sigma-Aldrich (St. Louis, MO, USA). The physiochemical quality characteristics of moisture, color, pH, titratable acidity (TA), acidity, salinity, amino-type nitrogen content, alcohol content, and reducing- and total-sugar content of *koji* were analyzed. Moisture content was measured using a moisture analyzer (WBA-110M, Daihan Scientific Co., Wonju, Korea). Color was determined using a color analyzer (CR-10 Plus, Konica Minolta, Tokyo, Japan). Titratable acidity and pH were analyzed on 10% (*w*/*v*) diluted *koji* with distilled water using a pH meter (Lab 850, SCHOTT Instruments, Deutschland, Germany). For determination of TA, the *koji* solution (10% w/v) was titrated using 0.1 M NaOH till pH 8.3. The amount of 0.1 M NaOH consumed (mL) was reported. Next, the acidity of *koji* was measured as follows: 1.5 g of *koji* was dissolved in 95% ethanol/ether (1:2, *v*/*v*) and stirred on a hot plate (SMHS-3, Daihan Scientific Co., Wonju, Korea) at a speed of 620 rpm for 20 min (SMHS-3, Daihan Scientific Co., Wonju, Korea). Then, 50 μL of 1% phenolphthalein solution was added and 0.1 N ethanol-potassium hydroxide (KOH) was used for titration. The acidity was determined by a visual assessment and the amount of KOH per gram of *koji* was calculated.

Salinity, amino-type nitrogen, and alcohol content were measured using 2% (*w*/*v*) diluted *koji* with distilled water. For salinity analysis, the *koji* solution was titrated following the Mohr method. For determination of amino-type nitrogen, the *koji* solution was extracted using a shaking water bath (MaCturdy-30, Daihan Scientific Co., Gangwon-do, Korea) at a speed of 120 rpm for an hour. Then, amino-type nitrogen was determined by the titration method using 0.1 N NaOH, until pH 8.4. After which, a 50 μL of 1% phenolphthalein solution was used as a color indicator, and the results were expressed as a percent per milligram (mg/%). To measure the alcohol content, the *koji* solution was filtered through Whatman no. 1 filter paper (Whatman International Ltd., Maidstone, England). Then, 1 mL of *koji* solution with 10 mL of 1 M CrO3 was mixed and let stand for 15 min. Absorbance was determined at 600 nm using a UV spectrophotometer (BioDrop, MET Laboratories, Inc., Baltimore, MD, USA). The standard curve and results were expressed in terms of the percentage of alcohol content. Finally, reducing- and total-sugar content were measured using 1% (*w*/*v*) diluted *koji* and distilled water. Reducing sugar was analyzed by filtering 1 mL of *koji* solution through Whatman no. 2 filter paper (Whatman International Ltd., Maidstone, England). Then, this *koji* solution and the DNS solution (3, 5-dinitrosalicylic acid) were mixed with 1 mL of distilled water with 1:1 ratio, and then it was heated at 100 °C for five minutes using a hot plate (SMHS-3, Daihan Scientific Co., Wonju, Korea) and immediately cooled on ice. The absorbance of each of test solution was recorded at 550 nm using a UV spectrophotometer (BioDrop, MET Laboratories, Inc., Baltimore, MD, USA). The standard curve and results were expressed in terms of glucose content (mg/g). Similarly, to measure the total sugar content, 1 mL of *koji* solution was mixed with of 5% phenol solution and 5 mL of 95% H_2_SO_4_ and put in room temperature for 20 min. The absorbance of each test solution was measured at 480 nm using a UV spectrophotometer (BioDrop, MET Laboratories, Inc., Baltimore, MD, USA). Glucose (mg/g) was used to record the standard curve and results.

### 2.3. Enzymatic Activity Analysis

Enzymatic activity, including amylase and protease activity, was measured following the method from a previous study [14,15], with minor modifications. For amylase activity, 3 g of *koji* with 30 mL of purified water were homogenized using a vortex mixer (VM-10, Daihan Scientific Co., Wonju, Korea) and extracted in a shaking water bath (MaCturdy-30, Daihan Scientific Co., Wonju, Korea) at a speed of 120 rpm for four hours. Then, centrifuged at a speed of 8279× *g* at 4 °C for 15 min (1580MGR, Gyrozen, Daejeon, Korea). The supernatant of the centrifuged *koji* solution was used as a crude enzyme solution to determine the amylase activity. To measure the α-amylase activity, 1 mL of *koji* solution was mixed with 2 mL of 1% starch solution at pH 7 using a 0.2 M sodium phosphate buffer and placed in a shaking water bath (MaCturdy-30, Daihan Scientific Co., Wonju, Korea) at 40 °C for 30 min. To halt the reaction of amylase, 10 mL of 0.1 N HCl was added and placed at room temperature for 30 min. Next, of an iodine solution was added and absorbance was recorded at 660 nm using a UV spectrophotometer (BioDrop, MET Laboratories, Inc., Baltimore, MD, USA). A unit of α-amylase activity was defined as the disappearance of an iodine binding 1% starch per minute in the assay reaction (unit/g). The β-amylase of the *koji* samples was measured as follows: 1 mL of *koji* solution and 1 mL of 0.5% starch solution with 0.4M sodium acetate buffer at pH 4.8 were thoroughly mixed in a shaking water bath (MaCturdy-30, Daihan Scientific Co., Wonju, Korea) at 30 °C for 30 min. Then, 2 mL of DNS solution (3, 5-dinitrosalicylic acid) was added and boiled at 100 °C for five minutes. Upon boiling, each *koji* solution was immediately cooled on ice and the absorbance was recorded at 550 nm using a UV spectrophotometer (BioDrop, MET Laboratories, Inc., Baltimore, MD, USA). A standard curve was made using a 0.2% maltose. The β-amylase activity was calculated according to the amount of maltose (mg) produced by 1 mL of the *koji* solution for a minute. To determine protease activity, 2 g of the *koji* sample with 40 mL of distilled water was mixed and filtered through Whatman no. 1 filter paper (Whatman International Ltd., Maidstone, England). Three ml of 0.6% casein solution (acidic: pH 2.6; neutral: pH 7.0) with 1 mL of *koji* solution was mixed and placed in a shaking water bath (MaCturdy-30, Daihan Scientific Co., Wonju, Korea) at 30 °C for 10 min. Then, 5 mL of 0.4 M trichloroacetic acid (TCA) solution was added to the *koji* solution and let stand for 20 min at 37 °C to halt the enzyme reaction. After which, the *koji* solution was centrifuged at a speed of 1147× *g* for five minutes (1580 MGR, Gyrozen, Daejeon, Korea). One ml of Folin-Ciocalteau reagent was added to 2 mL of *koji* supernatant solution and then 5 mL of 0.4 M Na_2_CO_3_ was mixed in. After 30 min, the absorbance at 660 nm was recorded using a UV spectrophotometer (BioDrop, MET Laboratories, Inc., Baltimore, MD, USA). Tyrosine (mg) was used to record a standard curve and a unit of protease activity was expressed based on the amount of tyrosine produced by 1 mL of the *koji* solution for one minute. All measurements were conducted in triplicates.

### 2.4. Descriptive Analysis

To determine the sensory characteristics of *koji* made with different kinds of raw materials, a descriptive analysis was conducted using a highly trained panel (*n* = 7) consisting of six females and one male, 22–38 years of age. Each panelist had more than 500 h of experience in sensory analysis of various food products using the Spectrum™ Method with a 15-point Universal scale. Thirty grams of *koji* were put in 70 mL white plastic cups (70 mm × 30 mm) and labelled with random three-digit codes. The *koji* samples used for sensory evaluation were prepared two hours prior to evaluation and kept at room temperature until testing. Panelists received four-2 h training sessions to calibrate the use of the 15-point Universal scale and develop the sensory lexicon for each *koji* type. Only the aroma attributes of the *koji* were evaluated. Panelists took three minutes of rest between the evaluation of each sample to minimize a carry-over effect. All analysis was conducted in triplicate. Panelists were invited to an appreciation dinner upon completion of the evaluation.

### 2.5. Statistical Analysis

The results of the physiochemical characteristics and enzymatic activities of the *koji* samples were expressed as the mean ± standard deviation of a triplicate analysis. A significant difference in the physiochemical and sensory characteristics of the *koji* types during the fermentation period was analyzed using a variance followed by Duncan’s multiple range test at a level of α = 0.05. A principal component analysis (PCA) was conducted to determine the correlation between the physiochemical quality characteristics and sensory characteristics of each type of *koji* using XLSTAT software from 2018 (Addinsoft, Paris, France). All statistical analysis was performed using SPSS (version 25.0; IBM Co., Amork, NY, USA), except for the PCA.

## 3. Results and Discussion

### 3.1. Comparison of the Physiochemical Quality Characteristics of koji

The physiochemical quality characteristics of soybean, rice, and wheat *koji* are shown in Table 1. Significant differences in several quality attributes, including pH, moisture content, a*, acidity, titratable acidity (TA), reducing- and total-sugar content, and alcohol content, were observed among the three *koji* samples (*p* < 0.05).

Soybean *koji* showed higher values in the moisture content, pH, a*, acidity, and TA compared to the other types of *koji* made with rice and wheat (*p* < 0.05). As for the moisture contents of *koji* samples, soybean *koji* had 61.8%, rice *koji* had 51.1%, and wheat *koji* had 26.9% respectively, and these values were significantly different to each other (*p* < 0.05). The reported moisture content of commercially-available *doenjang* was approximately 50–55% [14]. Compared to the reported moisture content of *doenjang*, wheat *koji* had significantly lower moisture content than other *koji* samples as well as moisture content of *doenjang.* The low moisture content in wheat *koji* might be attributed to the fact that wheat *koji* is prepared with wheat flour, which has a lower moisture content at the beginning of the *koji*-process compared to rice and soybean. It is worth to note that the moisture contents of soybean, rice and wheat *koji* in 0-h fermentation were 59%, 46.3%, and 26%, respectively. As seen in this study, the moisture content of the raw materials may have influenced the moisture content of the final *koji* products. The pHs of *koji* made with different ingredients were 6.2 (soybean *koji*), 5.5 (rice *koji*), and 5.7 (wheat *koji*), and no significant differences between samples were observed (*p* > 0.05). The pH ranges of *koji* were within the range of reported pHs in commercially-available *doenjang* samples, which correspond to 4.7–5.4 [14]. This is as expected because commercial *doenjang* making process is heavily dependent on *koji*, therefore the metabolites including organic acids in *koji* may have influenced the final pH of *doenjang*. Soybean *koji* had the highest values in TA and acidity. The results of the acidity measurements might be influenced by the presence of *Aspergillus oryzae*, which produces organic acid in the raw materials [16,17]. Salinity values of *koji* samples ranged from 8.2–10.3% and no significant difference was seen among samples (*p* > 0.05). No significant differences in amino-type nitrogen were also observed among the three *koji* samples. Previous studies reported an increase in amino-type nitrogen of about 0.7–1.5 mg/% over a 72 h fermentation period [11,17]. The level of amino-type nitrogen reported in this study for *koji* made with soybean, rice, and wheat is within the range of previously reported values. However, rice *koji* had the highest level in reducing- and total-sugar and alcohol contents followed by wheat and soybean *koji* (*p* < 0.05). Previous studies on the metabolite analysis of rice *koji* made with two different microorganisms (*Aspergillus oryzae* and *Bacillus amyloliquefaciens*) reported that rice *koji* made with *A. oryzae* has higher levels of sugars, sugar alcohols, and organic acids such as succinic, glyceric, fumaric, malic, kojic, citric, and gluconic acids [9]. These are the metabolites from carbohydrate metabolism. Therefore, the higher levels of reducing and total sugar in rice *koji* may be a result of the carbohydrate metabolism in rice, as most of the ingredients in rice *koji* are composed of carbohydrates. Soybean *koji* had the lowest level of sugar, which may be due to the high protein content in soybean. Soybean *koji* also had a low alcohol content and higher levels of acidity and TA compared to the other types of *koji* (*p* < 0.05). Previous studies reported that the early stage of fermentation is important for metabolite formation in *koji*, and that the production of alcohol occurs at an early stage in the fermentation process (between 0–24 h), while organic acids were produced at the end of the fermentation process [16,17].

### 3.2. Enzymatic Activity Results

The enzymatic activities of the three different types of *koji* are shown in Table 2. The enzymatic activities of *koji* depend on the characteristics of the raw materials, types of mold, fermentation time, and storage conditions [6,18,19]. Results of the amylase and protease activities in soybean, rice, and wheat *koji* were significantly different (*p* < 0.05). The α-amylase activities of soybean, rice, and wheat *koji* were 35.7 unit/g, 34.8 unit/g, and 36.3 unit/g, respectively, and the α-amylase activity of wheat *koji* was significantly higher than that of rice *koji* (*p* < 0.05). Similarly, rice *koji* showed higher β-amylase activities than the other two *koji* samples (*p* < 0.05). A high amylase activities of rice *koji* might be due to the nutritive value of rice [6], where rice is composed of more than 90% carbohydrates (mainly amylose) and less than 7% proteins, as a result of the milling process [20]. Higher content of amylase activities (α- and β-) may be due to the higher amount of amylose content in the raw materials (in this case, rice). Protease activity can be used to measure the hydrolysis ability of a protein. The results of the protease activities in soybean *koji* were significantly higher than the protease activities in rice and wheat *koji*, which is consistent with previous studies that reported low protease activities in rice and barley *koji* during the fermentation process [7,9,21]. Lower protease activities in rice and wheat *koji* may be due to the lower protein content in the raw materials (i.e., rice and wheat flour), which limits the protease expression during the fermentation process [7]. When compared to the acidic protease activities of *doenjang*, which is around 70–110 unit/g for commercially-made *doenjang* and 90–120 unit/g for traditionally-made *doenjang* [14], acidic protease activities of soybean *koji* was still lower than acidic protease activities of *doenjang*. Similarly, neutral protease activities ranged from 44–130 unit/g for commercially-made *doenjang*, while neutral protease activities of traditionally-made *doenjang* ranged from 88–120 unit/g; these values were higher than neutral protease activities of soybean *koji* reported in this study. This indicates that protease activities continuously increase during *doenjang* fermentation process, which explains the increased levels of protein-degradation compounds responsible for distinctive flavors in *doenjang* [1].

### 3.3. Descriptive Analysis Results

The sensory profiles of soybean, rice, and wheat *koji* were characterized using a trained panel (*n* = 7), as shown in Table 3. The intensity of the three different *koji* samples was assessed using a 15-point Universal scale. The sensory lexicon for describing the aromatic characteristics of soybean, rice, and wheat *koji* was defined as followings: bean sprout, balsamic, soy sauce, *cheonggukjang,* cracker, solvent, rice powder, parboiled rice, overripe banana, syrup, wood, and roasted. The definitions and references for each aroma characteristic are included in Table 3. Significant aroma differences were observed between the three *koji* samples (*p* < 0.05).

Soy sauce and solvent were commonly present aroma characteristics among the three types of *koji*. Compared with previous studies of the flavor of *doenjang*, the common sensory characteristics between *doenjang* and *koji* in the present study were balsamic, soy sauce, and solvent [22,23,24,25]. The characteristics of bean sprout, balsamic, *cheonggukjang*, and cracker were strongly identified in soybean *koji*. In particular, soybean *koji* had higher intensities of *cheonggukjang* and cracker aromatics compared to rice and wheat *koji*. Previous studies analyzing the flavor of *doenjang* using a descriptive analysis defined soybean-related characteristics as cooked soybean, roasted soybean, roasted bean/roasted nutty, and *cheonggukjang* [23,24,25]. The results from this study indicate a similarity in the aroma characteristics in soybean *koji* and *doenjang*, which is as expected because commercially-made *doenjang* typically utilizes soybean *koji*. Rice *koji* had higher characteristics in soy sauce and solvent aromatics than soybean and wheat *koji.* Rice *koji* also had higher sweetness-related characteristics, such as overripe banana and syrup, compared to the other *koji* samples (*p* < 0.05). As mentioned earlier, these sweet-related aroma characteristics may have been attributed from the carbohydrate metabolism of rice, as rice had higher content of starch than soybean and wheat. Combination of higher carbohydrate content and high amylase activities (α- and β-) may have influenced the sweet-like aroma characteristics of rice *koji*. The aromatics of rice powder were only identified in rice and wheat *koji*. The characteristics of wood and roasted were higher in wheat *koji* than the other *koji* samples (*p* < 0.05).

### 3.4. Correlating Physiochemical Quality Characteristics, Enzymatic Activities, and Descriptive Analyses Result

**Figure 1:**
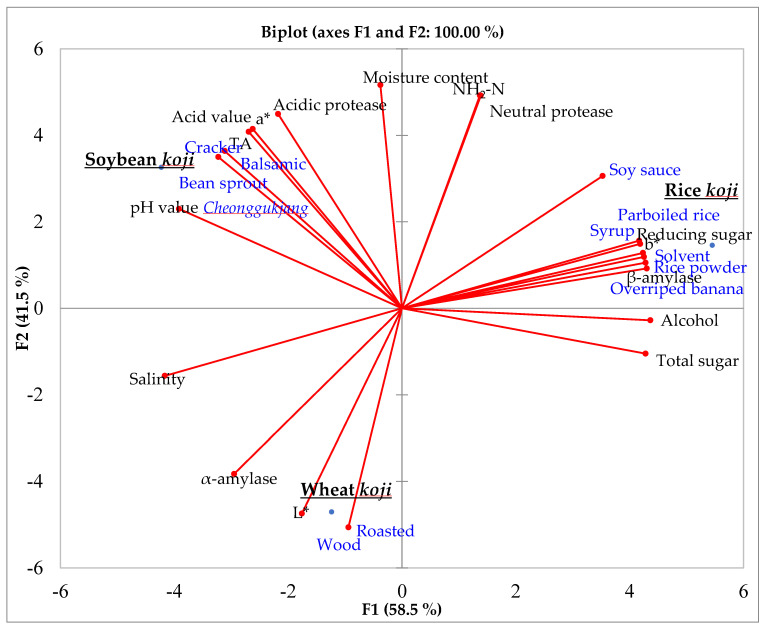
Principal Component Analysis Biplot of physiochemical and sensory characteristics of three *koji* made with different sources (soybean, rice and wheat).

A principal component analysis (PCA) biplot of the three types of *koji* based on physiochemical quality characteristics, enzymatic activities, and descriptive analyses can be found in Figure 1. A PCA biplot shows where each *koji* sample is located in a sensory and quality characteristics map.

In this map, soybean, rice, and wheat *koji* were differentiated by their quality, enzymatic, and sensory characteristics. Soybean *koji* showed a high correlation with the acidity, TA, pH, and acidic protease, and was characterized by balsamic, cracker, and *cheonggukjang* aromatics. This result is in agreement with what was stated in earlier in this paper. *Aspergillus oryzae* is known to produce hydrolytic enzymes, and thus the amino-acid degradation process from hydrolytic enzymes produced by *A. oryzae* may have influenced organic-acid and amino-acid production in soybean *koji* [7]. These high protease activities and consequent amino-acid degradation process in soybean *koji*, may have created the balsamic, bean sprout *cheonggukjang* and cracker aroma characteristics. Previous study reported that *Cheonggukjang*, and balsamic aromatics are typical aroma characteristics of traditionally-made *doenjang* in which have high protease and other enzymatic activities [25]. The finding from this study is in agreement with previously reported results. Rice *koji* showed a high correlation with sweetness-related attributes, such as reducing- and total-sugar content, alcohol content, and β-amylase. The carbohydrate metabolism from rice starch may have influenced the high levels of reducing and total sugars in rice *koji*, as well as the alcohol content. The presence of alcohol in rice *koji* may have influenced its aromatic characteristics, such as overripe banana and syrup. The characteristic aromatics of rice *koji* were rice powder, solvent, overripe banana, syrup, parboiled rice, and soy sauce. Previous studies also reported that *doenjang* samples high in total and reducing sugar showed a high correlation with sweetness aromatics [14]. The wheat *koji* sample was characterized by a high L* value and woody and roasted aromatics. The high L* value of wheat *koji* indicates a whiter color, which is a distinctive characteristic of wheat *koji* in comparison to the other types.

## 4. Conclusions

This study established the physiochemical quality characteristics, enzymatic activities, and sensory aromatic characteristics of soybean, rice, and wheat *koji*. Differences were observed in the three types of *koji* prepared with different raw materials. Soybean *koji* had high levels of pH, moisture, acidity, and TA, as well as soybean-related aromatics characterized by bean sprout, cracker, and *cheonggukjang*. Rice *koji* had high levels of total and reducing sugar as well as aromatics characterized by overripe banana, syrup, solvent, and parboiled rice. Wheat *koji* had a whiter color than the other samples, and woody and roasted aromatics. The differences between the three *koji* samples were determined by the different raw ingredient of each type.

## Figures and Tables

**Table 1:** Comparison of physiochemical characteristics of three different *koji* after 72 h fermentation.

		Soybean *koji*	Rice *koji*	Wheat *koji*	*p*-Value
Moisture content (%)		61.8 ± 0.9 ^a^	51.1 ± 4.3 ^b^	26.9 ± 1.3 ^c^	0.00
Color measurement	L*	8.8 ± 5.2	7.6 ± 2.7	13.4 ± 13.2	0.46
	a*	3.4 ± 1.2 ^a^	0.6 ± 2.0 ^b^	0.0 ± 2.0 ^b^	0.01
	b*	4.4 ± 2.0	6.8 ± 1.6	4.6 ± 1.9	0.08
pH value		6.2 ± 0.8 ^a^	5.5 ± 0.2 ^b^	5.7 ± 0.3 ^a, b^	0.08
Titratable acidity (mL)		3.2 ± 1.7 ^a^	0.9 ± 0.1 ^b^	0.8 ± 0.4 ^b^	0.00
Acid value (KOH/g)		21.4 ± 4.0 ^a^	6.7 ± 1.7 ^b^	3.1 ± 0.4 ^c^	0.00
Salinity (%)		10.0 ± 0.4	8.2 ± 0.2	10.3 ± 0.3	0.54
NH_2_-N (mg/%)		1.1 ± 1.4	1.2 ± 0.8	1.3 ± 0.4	0.22
Alcohol (g/%)		3.8 ± 1.2 ^b^	5.2 ± 0.8 ^a^	4.3 ± 0.7 ^a, b^	0.06
Reducing sugar (mg/g)		13.9 ± 1.8 ^b^	90.3 ± 43.1 ^a^	15.2 ± 5.1 ^b^	0.00
Total sugar (mg/g)		16.8 ± 11.7 ^c^	107.5 ± 10.9 ^a^	61.1 ± 5.7 ^b^	0.00

Numbers represent mean ± standard deviation of triplicate analyses of each physiochemical characteristics. Numbers in a row that does not share same alphabetical letter represent significant differences at α = 0.05.

**Table 2:** Enzymatic activities of three different *koji* after 72 h fermentation.

Enzymatic Activities	Soybean *koji*	Rice *koji*	Wheat *koji*
α-amylase (unit/g)	35.7 ± 0.6 ^a, b^	34.8 ± 0.5 ^b^	36.3 ± 0.4 ^a^
β-amylase (unit/g)	48.3 ± 4.4 ^b^	75.1 ± 2.4 ^a^	52.0 ± 8.0 ^b^
Acidic protease (unit/g)	24.0 ± 5.4 ^a^	8.5 ± 5.2 ^b^	1.5 ± 1.1 ^b^
Neutral protease (unit/g)	13.5 ± 1.8 ^a^	15.1 ± 2.4 ^a^	1.2 ± 2.2 ^b^

All results of enzymatic activities were represented as mean ± standard deviation. The alphabetic letter in the row for each *koji* represented a significant difference (*p* < 0.05). Numbers in a row that does not share same alphabetical letter represent significant differences at α = 0.05.

**Table 3:** Descriptive sensory analysis about three *koji* samples using a trained panel (*n* = 7).

Term	Definition	Soybean *koji*	Rice *koji*	Wheat *koji*
Bean sprout	The aromatics associated with bean sprout (Ref: steaming bean sprout in water for 20 min)	0.07 ^a^	0.00 ^a^	0.00 ^a^
Balsamic	The aromatics associated with balsamic vineager (Ref: balsamic vinegar, Ottugi, Anyang, Gyeonggi-do, Korea)	0.20 ^a^	0.00 ^a^	0.00 ^a^
Soy sauce	The aromatics associated with commercially-made soy sauce (Ref: 501 soy sauce, Sempio^®^, Icheon, Gyeonggi-do, Korea)	0.86 ^b^	1.67 ^a^	0.47 ^b^
*Cheonggukjang*	The aromactics associated with *cheonggukjang* (Ref: Cheonggukjang, Pulmuone, Iksan, Jellabuk-do, Korea)	1.95 ^a^	0.00 ^b^	0.00 ^b^
Cracker	The aromatics associated with unsalted cracker (Ref: Cham-cracker, Crown, Deajeon, Korea)	0.40 ^a^	0.00 ^b^	0.00 ^b^
Solvent	The aromatics associated with chemical refrigerator	0.27 ^b^	1.73 ^a^	0.36 ^b^
Rice powder	The aromatics associated with rice powder (Ref: Organic rice powder, Momsrice, Gwangju, Gyeonggi-do, Korea)	0.00 ^a^	0.06 ^a^	0.01 ^a^
Parboiled rice	The aromatics associated with parboiled rice (Ref: Instant rice without heating, CJ Cheil-jedang, Gangneung, Gangwon-do, Korea)	0.00 ^a^	0.07 ^a^	0.00 ^a^
Overriped banana	The aromatics associated with overly-ripped banana (Ref: overly-riped banana)	0.00 ^b^	1.83 ^a^	0.00 ^b^
Syrup	The aromatics associated with maple syrup (Ref: maple syrup, L.B. Maple Treat Corp, Quebec, Canada)	0.00 ^b^	1.18 ^a^	0.00 ^b^
Wood	The aromatics associated with wet wood (Ref: hot steaming wet sauna)	0.00 ^b^	0.00 ^b^	0.47 ^a^
Roasted	The aromatics associated with popcorn (Ref: Lohi popcorn, Sajo, Yeongcheon, Gyeongsangbuk-do, Korea)	0.00 ^b^	0.00 ^b^	2.31 ^a^

Numbers represent mean of intensities of each term rated by highly trained panel (*n* = 5) using 15-pt Universal scale. Numbers in a row that does not share same alphabetical letter represent significant differences at α = 0.05.
